# Clinical and positron emission tomography responses to long-term high-dose interferon-α treatment among patients with Erdheim–Chester disease

**DOI:** 10.1186/s13023-018-0988-y

**Published:** 2019-01-10

**Authors:** Xin-xin Cao, Na Niu, Jian Sun, Hao Cai, Feng-dan Wang, Yi-ning Wang, Ming-hui Duan, Dao-bin Zhou, Jian Li

**Affiliations:** 1 Department of Hematology, Peking Union Medical College Hospital, Chinese Academy of Medical Sciences & Peking Union Medical College, 1 Shuai Fu Yuan Hu Tong, Dongcheng District, Beijing, 100730 China; 2 Department of Nuclear Medicine, Peking Union Medical College Hospital, Chinese Academy of Medical Sciences & Peking Union Medical College, Beijing, China; 3 Department of Pathology, Peking Union Medical College Hospital, Chinese Academy of Medical Sciences & Peking Union Medical College, Beijing, China; 4 Department of Radiology, Peking Union Medical College Hospital, Chinese Academy of Medical Sciences & Peking Union Medical College, Beijing, China

**Keywords:** Erdheim–Chester disease, *BRAF*^*V600E*^ mutation, Interferon-α, Positron-emission tomography

## Abstract

**Background:**

Erdheim–Chester disease (ECD) is a rare multi-systemic form of histiocytosis. Treatment with BRAF inhibitors has markedly improved outcomes of ECD; however, this targeted therapy is expensive (estimated annual cost is $50,000). Since estimated annual cost of interferon-α (IFN-α) is only approximately $1600 in China, we retrospectively evaluated the long-term therapeutic efficacy of IFN-α and the value of 18F-fluorodeoxyglucose positron emission tomography (FDG-PET) as an assessment method among 32 ECD patients who received high dose IFN-α therapy at Peking Union Medical College Hospital.

**Results:**

The median age at diagnosis was 48 years (range, 6–66 years). The median duration of treatment was 18.5 months (range, 1–51 months). The overall clinical response rates were 80.0%, including 33.3% complete response, 36.7% partial response and 10.0% stable disease. Thirty-one patients underwent a total of 81 scans by FDG-PET. Seventeen patients had serial FDG-PET results, nine patients had experienced a partial metabolic response at the last follow-up. The median reduction of ratios between the most active target lesion standardized uptake value (SUV) and liver SUV from baseline to last FDG-PET scan was 61.4% (range, 8.8–86.6%). Eight of thirteen patients who experienced continuous clinical improvement during follow-up had at least one target lesion SUV increased by FDG-PET which decreased in subsequent scans without changing treatment strategy. The estimated 3-year progression-free survival (PFS) and overall survival (OS) were 64.1 and 84.5%, respectively. Central nervous system (CNS) involvement was the only predictor for poor PFS and OS.

**Conclusions:**

High-dose IFN-α treatment is a cost-effective option, especially for patients without CNS involvement. Single target lesion SUV elevation according to FDG-PET do not accurately demonstrate disease progression, but serial FDG-PET imaging effectively discriminate treatment response.

## Introduction

Erdheim–Chester disease (ECD) is a rare multi-systemic form of histiocytosis that is characterized by infiltration of lipid-laden foamy macrophages into different tissues. The clinical spectrum of ECD ranges from asymptomatic to life-threatening multi-organ involvement, as pathologic histiocytes can infiltrate virtually every organ and tissue [1, 2].

Recurrent somatic activating mutations of *BRAF*^*V600E*^ are found in 57% of archived Langerhans cell histiocytosis (LCH) lesions [3] and in 50–70% of infiltrating histiocytes sampled from ECD lesions [2, 4]. The concomitant occurrence of LCH and ECD (mixed histiocytosis) is not fortuitous and may linked to *BRAF*^*V600E*^ mutation [5]. Since 2015, the use of the BRAF inhibitor vemurafenib has changed the initial treatment approach in 50–70% of patients with ECD [6]. However, the optimum duration of treatment with vemurafenib remains unknown, and 75% of patients relapse after stopping this targeted therapy [7]. As a consequence, treatment might need to be continued until either disease progression or intolerable adverse effects develop [8]. In addition, the estimated annual wholesale cost of vemurafenib is approximately 50,000 dollars in China, which is far beyond what most patients in low-income countries can afford. In an effort to looking for a cost-effective treatment option, we retrospective review the outcomes of Interferon-α (IFN-α) therapy among ECD patients in our center.

Historically, IFN-α has been used with variable efficacy as a treatment for ECD [9, 10]. The estimated annual cost of IFN-α is approximately 1600 dollars in China, which makes IFN-α still the first treatment option for ECD patients in low-resource countries [11]. However, the long-term outcomes of IFN-α therapy are unclear. Moreover,treatment evaluation of ECD is difficult given the rarity of this condition and the fact that it can affect multiple organs. 18F-fluorodeoxyglucose positron emission tomography (FDG-PET) scan was reported to be a good indicator of disease activity [12]. FDG-PET has been used to evaluate the overall therapeutic response of vemurafenib [6]. However, the role of serial FDG-PET imaging on clinical decision making in ECD patients treated with IFN-α had never been investigated.

The aim of the current study was to describe the long-term treatment outcomes and efficacy of FDG-PET for evaluating therapeutic responses among a cohort of ECD patients who were treated with IFN-α at Peking Union Medical College Hospital.

## Methods

### Patients

A retrospective review was conducted among patients who were diagnosed with ECD and had received high-dose IFN-α therapy for at least 1 month at Peking Union Medical College Hospital between January 2010 and May 2018. Diagnosis of ECD was based on typical clinical presentation, radiologic presentation, and histologic findings that were reviewed independently by two pathologists. Mixed histiocytosis (ECD & LCH) was diagnosed as previously described [5]. Informed consent was obtained from all patients and the protocol was approved by Peking Union Medical College Hospital Ethics Committee. The present study was performed in accordance with the ethical standards of the 1964 Declaration of Helsinki and its later amendments.

### Clinical, laboratory, imaging, and genetic data

Clinical data were collected regarding age, sex, lesion location, physical examination, routine biologic analysis, treatment, and survival. Serum level of the cytokines interleukin (IL)-6, IL-8, IL-10, and tumor necrosis factor (TNF)-α were measured by the electrochemiluminescence immunoassay (SIEMENS Immulite 1000). Imaging data were collected from FDG-PET, thoracic and abdominal computed tomography (CT), and cardiac and cerebral magnetic resonance imaging (MRI). The presence of the *BRAF*^*V600E*^ mutation was detected by pyrosequencing or immunohistochemistry as previously described [2].

### Treatment

High-dose IFN-α therapy was defined as the subcutaneous administration of either 600 MIU or 900 MIU of IFN-α, three times per week. Patients continued to receive IFN-α until two independent hematologists confirmed disease progression, intolerable adverse effects developed or the patient wished to stop treatment.

### Response criteria

All patients were followed up every 3–6 months. Clinical responses were categorized as follows: (1) complete response (CR): complete resolution of symptoms attributed to ECD; (2) partial response (PR): partial resolution of symptoms attributed to ECD; (3) stable disease (SD): no change in symptoms attributed to ECD; or (4) progressive disease (PD): worsening of symptoms attributed to ECD [13].

Activity of ECD was evaluated based on the clinical response and CT, MRI and FDG-PET imaging changes at various sites of ECD involvement. Organ involvement was assessed by FDG-PET. MRI was used to detect CNS and cardiac lesions. Enhanced CT was used to assess vascular involvement.

Disease progression was defined as 1) clinical PD or new organ involvement detected by CT, MRI or FDG-PET or 2) clinical SD together with a minimum 30% increase in standardized uptake value (SUV) of target lesions confirmed by two separate FDG-PET scans taken at least 3 months apart.

Target lesions were defined as the most active lesion measured by SUV on FDG-PET before treatment. A second active lesion that could be followed on successive FDG-PET examinations was also studied for each patient. We used the ratios between the first (max 1) and second (max 2) most active target lesion SUV and liver SUV (SUV_max1_/SUV_liver_ and SUV_max2_/SUV_liver_) for follow-up, to eliminate heterogeneity.

### Data analysis

The Fisher exact test was used to compare categorical variables, whereas the Mann–Whitney test was used to compare continuous variables between groups. Overall survival (OS) was defined as the time from diagnosis to the date of death or last follow-up. Progression-free survival (PFS) was calculated from the date of diagnosis until the date of disease progression, relapse, or death from any cause. Kaplan–Meier analysis was used to assess survival analysis, with the survival curves compared using the log-rank test. We performed all statistical analyses using SPSS version 21 software (IBM Corp., Armonk, NY, USA), and considered *P*-values of less than 0.05 to be statistically significant.

## Results

### Characteristics of the patients

A total of 32 patients (16 male and 16 female) met the inclusion criteria. Four of them were diagnosed with mixed ECD and LCH. The median age at diagnosis was 48 years (range, 6–66 years).

The demographic and clinical characteristics of the patients are presented in Table 1. The median number of organs involved was 4 (range 1–8). The main sites of involvement were the bones (93.8%), retroperitoneum (40.6%), lungs (37.5%), vasculature (37.5%), central nervous system (CNS, 34.4%), pericardium (28.1%), pleura (21.9%), skin (18.8%), pituitary (15.6%), heart (12.5%), retro-orbital involvement (12.5%), nerve root (9.4%), muscles (6.3%), thymus (6.3%), thyroid (3.1%) and breasts (3.1%).

**Table 1:** Demographic and clinical characteristics of the ECD patients according to their BRAF status

Characteristic	Total cohort (*n* = 32)	*BRAF*^*V600E*^ mutation (*n* = 21)	BRAF WT (*n* = 9)	*P* value
Age at diagnosis, years (median, range)	48 (6–66)	52 (6–66)	37 (32–56)	NS
Number of involved organs (median, range)	4 (1–8)	5 (2–8)	2 (1–7)	NS
organs of involvement				
Bone	30 (93.8%)	20 (95.2%)	8 (88.9%)	NS
Retroperitoneum	13 (40.6%)	10 (47.6%)	2 (22.2%)	0.193
Lungs	12 (37.5%)	9 (42.9%)	2 (22.2%)	NS
Vasculature	12 (37.5%)	10 (47.6%)	1 (11.1%)	0.057
Central nervous system	11 (34.4%)	8 (38.1%)	3 (33.3%)	NS
Pericardium	9 (28.1%)	8 (38.1%)	1 (11.1%)	0.139
Pleura	7 (21.9%)	7 (33.3%)	0 (0.0%)	0.048
Skin	6 (18.8%)	4 (19.0%)	2 (22.2%)	NS
Pituitary	5 (15.6%)	4 (19.0%)	1 (11.1%)	NS
Heart	4 (12.5%)	2 (9.5%)	1 (11.1%)	NS
Exophthalmos	4 (12.5%)	3 (14.3%)	1 (11.1%)	NS
Nerve roots	3 (9.4%)	1 (4.8%)	1 (11.1%)	NS

*WT* wild type, *NS* not statistically significant

Two patients did not have an adequate amount of tissue available for genomic testing. *BRAF*^*V600E*^ mutations were detected among 18/25 patients (72.0%) using PCR. Immunohistochemical analysis revealed positive staining for *BRAF*^*V600E*^ for 6/13 patients (46.2%). Finally, we confirmed that 21/30 patients (70.0%) had a *BRAF*^*V600E*^ mutation. Clinical presentation according to *BRAF*^*V600E*^ status is listed in Table 1. Patients with the *BRAF*^*V600E*^ mutation had more pleura involvement than did BRAF WT patients (*p* = 0.048). The data hint at a trend of more vascular, pericardial and retroperitoneal involvement in the *BRAF*^*V600E*^ mutation group, but these differences did not reach statistical significance.

At baseline, 14 patients (43.8%) had an elevated platelet count. Twenty-three patients (71.9%) had elevated serum high sensitive C-reactive protein (hsCRP) levels. The erythrocyte sedimentation rate (ESR) level was elevated among 20/26 (76.9%) patients. Fibrinogen levels were elevated among 22/26 (84.6%) patients. Elevated serum IL-6 levels were found among 18/23 (78.3%) patients (Table 2). Elevated serum IL-8 levels were found among 10/21 (47.6%) patients. Elevated serum TNF α levels were detected among 20/22 patients (90.9%). None of these 21 patients had elevated IL-10 levels.

**Table 2:** Level of serum cytokines IL-6, IL-8 and TNF-α at baseline

	Median (pg/mL)	Range (pg/mL)	Normal range (pg/mL)
IL-6 level	15.7	3.1–95.8	< 5.9
IL-8 level	54	5–755	< 62
TNF-α level	17.1	6.6–208.0	< 8.1

### Treatment and clinical response

A total of 26 (81.3%) patients received IFN-α as the first-line treatment. One (3.1%) patient with the *BRAF*^*V600E*^ mutation received vemurafenib after 3 months of treatment with IFN-α. Three (9.4%) patients who had mixed ECD and LCH received 6 courses of methotrexate (1 g/m^2^ on day 1) and cytarabine (100 mg/m^2^/d for 5 days) and then received IFN-α (600 MIU 3 times/week) for maintenance. One (3.1%) patient who had CNS and bone lesions received four courses of cytarabine 500 mg/m^2^ every 12 h for 3 days and then received IFN-α (600 MIU 3 times/week) for maintenance. Two (6.2%) patients received glucocorticoids as first-line therapy and received IFN-α after disease progression.

The median duration of follow-up was 24 months (range, 1–51 months). The median duration of IFN-α therapy was 18.5 months (range, 1–51 months). One patient stopped IFN-α treatment after 8 months owing to the development of anorexia and asthenia. Clinical responses were available for 30 of the 32 patients because two patients had received IFN-α for less than 3 months. The clinical response rates were as follows: CR, 33.3% (*n* = 10); PR, 36.7% (*n* = 11); SD, 10.0% (*n* = 3); and PD, 20.0% (*n* = 6).

### Laboratory evaluation during treatment

**Fig. 1:**
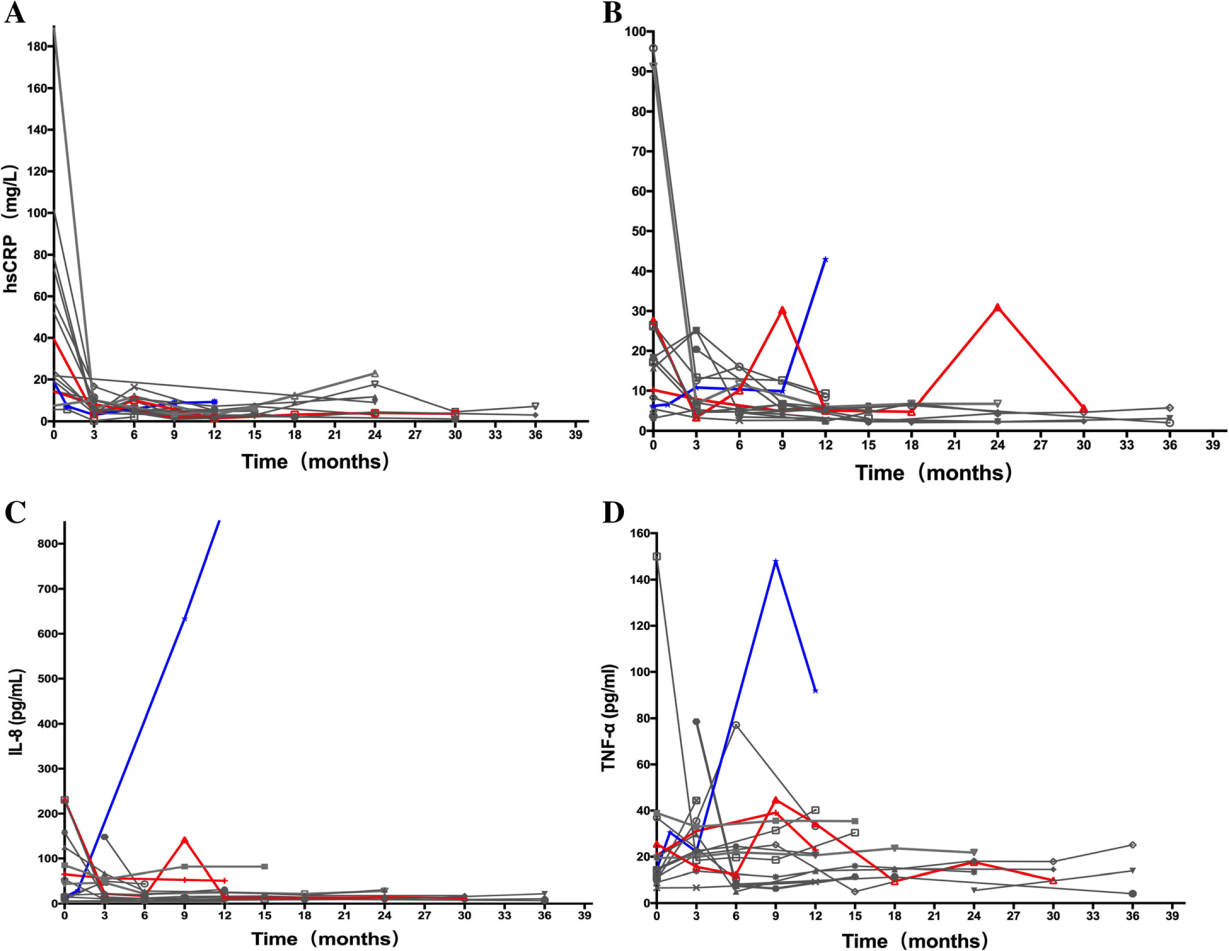
Changes in laboratory measures during treatment. **a**. Serum high sensitive CRP (hsCRP) levels; **b** Serum cytokines interleukin-6 (IL-6) levels; **c** Serum interleukin-8 (IL-8) levels; D. Serum tumor necrosis factor-α (TNF-α) levels. In all panels, the red lines represent patients who experienced disease progression during treatment. The blue lines represent patients who died during treatment. The gray lines represent patients who experienced continuous clinical improvement

Eighteen of 32 patients had serial results of hsCRP, IL-6, IL-8 and TNFα levels. Changes in the laboratory measures during treatment are shown in Fig. 1. Two of 18 patients experienced disease progression (red line in Fig. 1). One had elevated hsCRP levels, 1/2 had elevated IL-6 levels, 1/2 had elevated IL-8 levels, and 2/2 had elevated TNFα levels. One male patient died as a result of disease progression (blue line in Fig. 1): his serum levels of IL-6, IL-8 and TNFα increased over the time course. As shown in Fig. 1 (gray line), 14 of 18 patients displayed continuous clinical improvement during the follow-up period (CR = 9; PR = 5). At least one of the laboratory measures had increased by greater than 30% among 12 of these 14 patients. Without changing treatment, the level of elevated laboratory measures decreased at the subsequent follow-up.

### FDG-PET follow-up

**Fig. 2:**
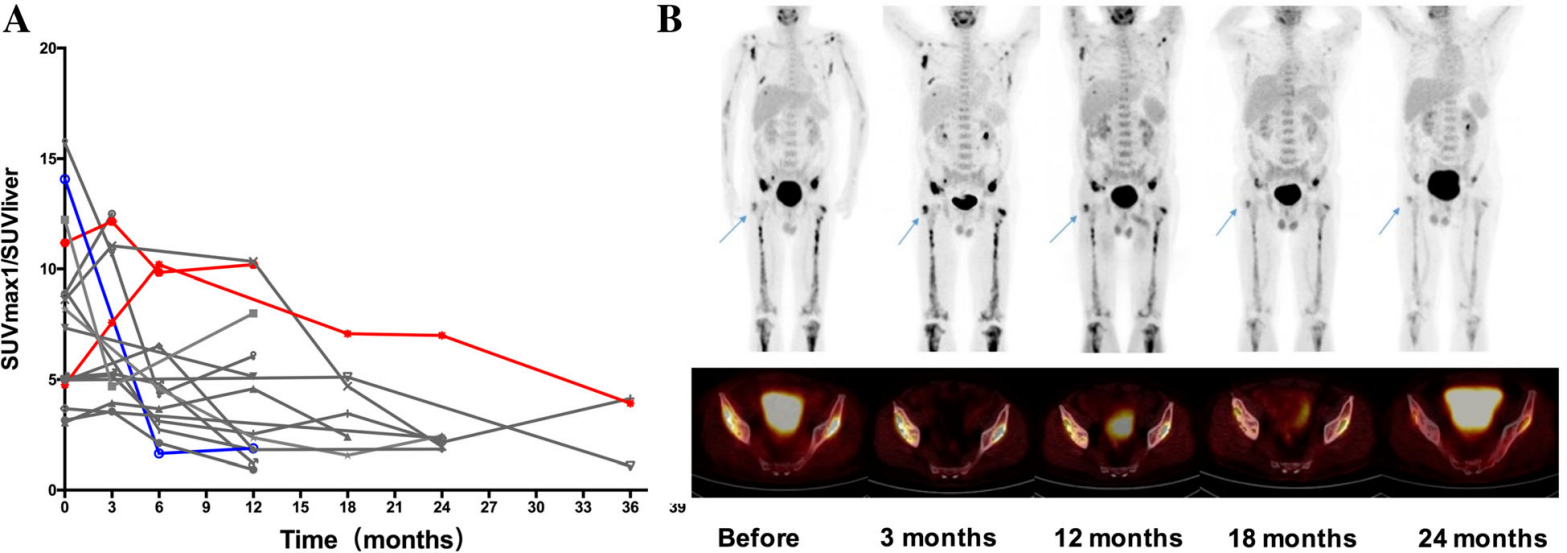
The baseline and follow-up FDG-PET scans showed changes in SUV_max1_/SUV_liver_ during treatment. **a** Serial FDG-PET scans of 17 patients. The red lines represent patients who experienced disease progression during treatment. The blue lines represent patients who died during treatment. The gray lines represent patients who experienced continuous clinical improvement. **b** Serial FDG-PET scans of one patient who experienced continuous clinical improvement. The most active target lesion of the patient at baseline was right ilium (arrow). Compared with baseline, SUV _max1_/SUV_liver_ increased at 3 months and 12 months after treatment. Without changing treatment strategy, SUV _max1_/SUV_liver_ decreased at 18 months and 24 months after treatment

**Fig. 3:**
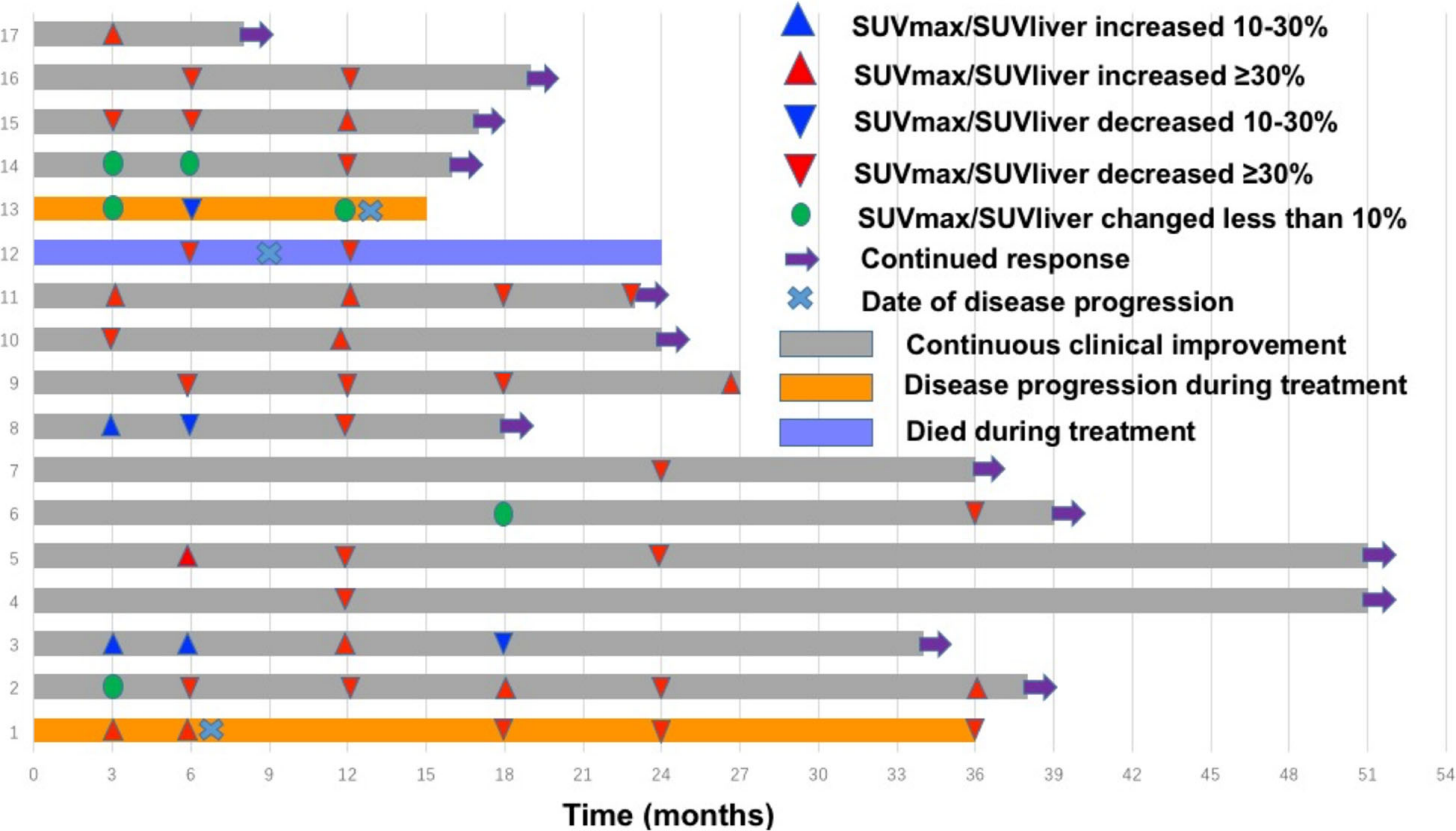
Efficacy of interferon-α treatment among a subgroup of patients with ECD (*n* = 17)

In all, 31 patients underwent a total of 81 FDG-PET scans. For each patient, we chose the two most active lesions as targets lesions. At recruitment, 30 patients underwent FDG-PET scans before treatment. The most active target lesions at baseline were bone (35/60, 58.3%), followed by CNS (*n* = 9, 15.0%), pleura (*n* = 3, 5.0%), nerve root (*n* = 3, 5.0%), muscle (*n* = 3, 5.0%), skin (*n* = 2, 3.3%), and heart, retroperitoneal, pericardium, thymus and vasculature (*n* = 1 each, 1.7%). A total of 17 patients underwent at least one follow-up FDG-PET scan, median of 4 [2–6]. The SUV_max1_/SUV_liver_ changed during treatment (Fig. 2a). At the last follow-up, nine (52.9%) of these patients had experienced a partial metabolic response. The median reduction in SUV_max1_/SUV_liver_ from baseline to last FDG-PET scan was 61.4% (range, 8.8–86.6%). In all, 13/17 (76.5%) patients experienced continuous clinical improvement during follow-up. Eight of 13 (61.5%) patients recorded at least one SUV_max1_/SUV_liver_ increase during follow-up: the median increase in this ratio was 32.0% (range, 2.6–45.4%). Figure 2b showed serial FDG-PET of one of these patients. Since none of them had clinical symptoms worsen, these 8 patients continued to received IFN-α therapy, and the SUV_max1_/SUV_liver_ according to the subsequent FDG-PET scans decreased (Fig. 3).

### Survival

**Fig. 4:**
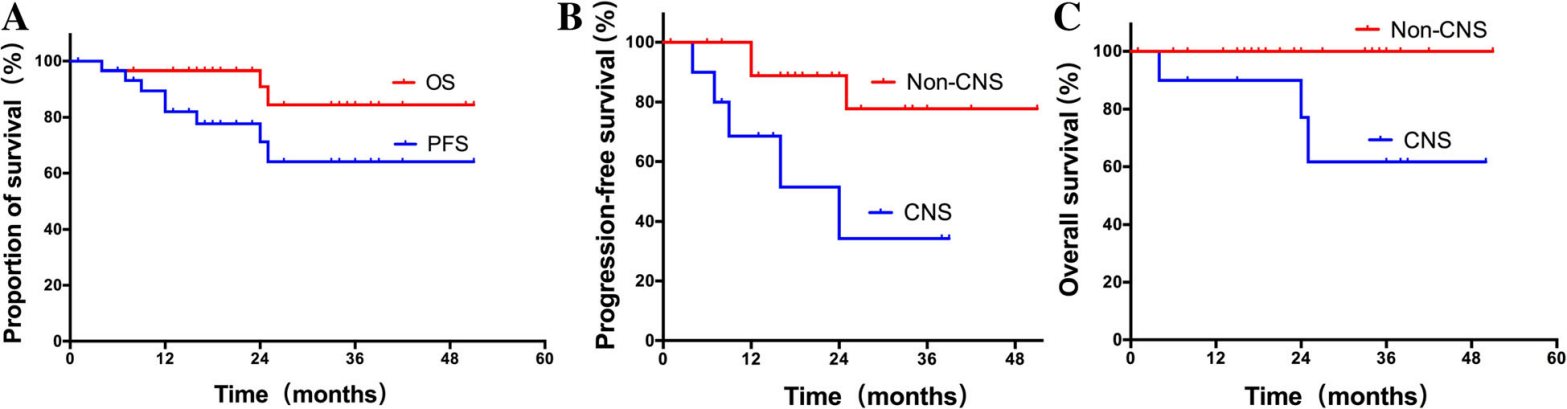
Survival for the whole cohort (*n* = 32). **a** PFS and OS. **b** Comparison of PFS according to CNS involvement. **c** Comparison of OS according to CNS involvement

Of the 32 patients included in the present study, three (9.4%) patients died and 8 (25.0%) experienced disease progression during follow-up. The estimated 3-year PFS and OS were 64.1 and 84.5%, respectively (Fig. 4a). The survival analyses showed that a significantly higher PFS was attained in patients without CNS involvement compared to those with CNS involvement (not reached vs 24 m, *p* = 0.018) (Fig. 4b). As shown in Fig. 4c, patients without CNS involvement also had a significantly higher OS than those with CNS involvement (*p* = 0.023). BRAF status was not an independent prognostic factor for PFS or OS.

## Discussion

ECD is a rare non-Langerhans cell histiocytosis. In this study, we identified a pleura phenotype closely linked to the BRAF status. We also found a trend of more vascular, pericardial and retroperitoneal involvement in the *BRAF*^*V600E*^ mutation group, as previous reported [14]. This indicated BRAF mutation status may denotes differences in disease presentation for patients with ECD.

Despite significant advances in our understanding of ECD, a standard treatment strategy is missing. Vemurafenib use in patients with *BRAF*^*V600E*^ mutation have increased antitumor efficacy [6]. Other targeted therapies including MEK inhibitor [7] and anti-IL-6 agent tocilizumab [15] seem promising, but with limited experience. Anakinra may be used in combination with kinase inhibitors and is effective in some cases characterized by difficult involvement [16–18]. IFN-α has been used with variable efficacy as a treatment for ECD and long-term outcomes of IFN-α therapy are unclear. Our study is one of the largest cohorts to date evaluating the role of high-dose IFN-α therapy as a treatment for ECD. We demonstrate that IFN-α has favorable clinical efficacy among patients with ECD, even for the most severe cases with multiple organ involvement. The overall clinical response rate was 80.0%, with an estimated 3-year PFS and OS of 64.1% and 84.5%, respectively. These outcomes are much better than those of other non-targeted therapies for ECD (overall clinical response rate of methotrexate [13], cladribine [19] and anakinra [20] were 23%, 52% and 50% separately). And only one patient stopped IFN-α treatment due to intolerable adverse effects. Together with the annual cost of IFN-α is only approximately 1600 dollars, IFN-α should still be the first treatment option for ECD patients in low-income countries.

In the present retrospective study, we found that FDG-PET was able to globally depict both the extent and the activity of the ECD lesions. The sensitivity varied greatly among the different sites of involvement. We found FDG-PET scanning was very helpful in assessing bone and CNS involvement in ECD. The sensitivity for detecting cardiovascular involvement was low when compared with that of MRI and CT scans. In this study, the vasculature, pericardium and heart involvement is approximately 40%, 30% and more than 10% separately detected by MRI or CT scans. However, the 60 most active lesions detected by FDG-PET only included one cardiac lesion, one pericardium lesion and one vascular lesion.

Although treatment with vemurafenib can promote a significant and fast FDG-PET response [6, 8], ECD remains a slowly evolving histiocytosis. Consequently, use of non-targeted therapies, such as IFN-α, usually leads to partial remission rather than complete recovery. The mechanism of IFN-α therapy is thought to induce immune-mediated histiocyte killing and the terminal differentiation of immature histiocytes, which is like tumor immunotherapy and quite different from that of cytotoxic chemotherapy or targeted therapy. The best documentation of the mechanism of action of tumor immunotherapy has been achieved by analyzing serial biopsies of regressing metastases after treatment with anti-CTLA4 antibodies among melanoma patients; this demonstrated that some patients treated with anti-CTLA4 antibodies experienced increased objective tumor burden and/or new lesions before a response was obtained [21, 22]. Therefore, there is a different set of response assessment criteria for tumor immunotherapy in solid tumors. However, it is unknown whether the same situation applies to ECD patients treated with IFN-α. We found that eight of 13 patients who experienced continuous clinical improvement had at least one target lesion SUV increased during follow-up. This elevation could be greater than 40%. While these patients achieved durable clinical improvement and lesion SUV of their subsequent FDG-PET decreased without further changing treatment strategy, calling into question the value of single target lesion SUV elevation according to FDG-PET to demonstrating disease progression. Consequently, new response assessment criteria might be required for IFN-α or other immunotherapeutic approaches for ECD.

Patients with ECD have been reported to have elevated levels of IFN-α, IL-12, chemokine ligand 18 (CCL18) and monocyte chemotactic protein-1 but decreased levels of IL-4 and IL-7; however, a previous study failed to demonstrate that the cytokine levels were related to disease activity [23, 24]. To our knowledge, this study is the largest series reviewing series change of cytokine levels during the same treatment. We measured the levels of hsCRP, IL-6, IL-8 and TNFα every 3–6 months among 18 patients and demonstrated that the levels of these molecules decreased after treatment but increased at disease progression. However, like the value of single target lesion SUV elevation according to FDG-PET, one single elevation of cytokine levels is not enough to demonstrating disease progression.

ECD is a heterogeneous disease with a variable prognosis. Previous studies have reported that CNS involvement and IFN-α treatment are independent prognostic factors [10, 25]. In this study, we demonstrated that CNS involvement was associated with poor survival among IFN-α treated patients. BRAF status was not an independent prognostic factor. This finding indicates that ECD patients with CNS involvement should seek new treatment strategies, such as targeted therapy.

The main limitation of our study is that it is a single-institution retrospective study, which might limit the generalizability of our results. However, all the records were reviewed independently by two hematologists to minimize bias or errors in data collection.

## Conclusion

In conclusion, high-dose IFN-α is a cost-effective treatment option, especially for patients without CNS involvement. We believe that single target lesion SUV elevation according to FDG-PET do not accurately demonstrate disease progression, but serial FDG-PET imaging effectively discriminate treatment response.

## Abbreviations

CNS: Central nervous system
CR: Complete response
CT: Computed tomography
ECD: Erdheim–Chester disease
FDG-PET: 18F-fluorodeoxyglucose positron emission tomography
IFN-α: Interferon-α
IL: Interleukin
LCH: Langerhans cell histiocytosis
MRI: Magnetic resonance imaging
OS: Overall survival
PD: progressive disease
PFS: Progression-free survival
PR: Partial response
SD: Stable disease
SUV: Standardized uptake value
TNF: Tumor necrosis factor
